# On the Rational Design of Core/(Multi)-Crown Type-II Heteronanoplatelets

**DOI:** 10.1021/jacs.3c00999

**Published:** 2023-05-09

**Authors:** Savas Delikanli, Betul Canimkurbey, Pedro Ludwig Hernández-Martínez, Farzan Shabani, Ahmet Tarik Isik, Ilayda Ozkan, Iklim Bozkaya, Taylan Bozkaya, Furkan Isik, Emek Goksu Durmusoglu, Merve Izmir, Hakan Akgun, Hilmi Volkan Demir

**Affiliations:** † Department of Electrical and Electronics Engineering, Department of Physics, UNAM − Institute of Materials Science and Nanotechnology, Bilkent University, Ankara 06800, Turkey; ‡ Luminous! Center of Excellence for Semiconductor Lighting and Displays, School of Electrical and Electronic Engineering, Division of Physics and Applied Physics, School of Physical and Mathematical Sciences, School of Materials Science and Engineering, Nanyang Technological University, Singapore 639798, Singapore; § Serefeddin Health Services Vocational School, Central Research Laboratory, Amasya University, Amasya 05100, Turkey

## Abstract

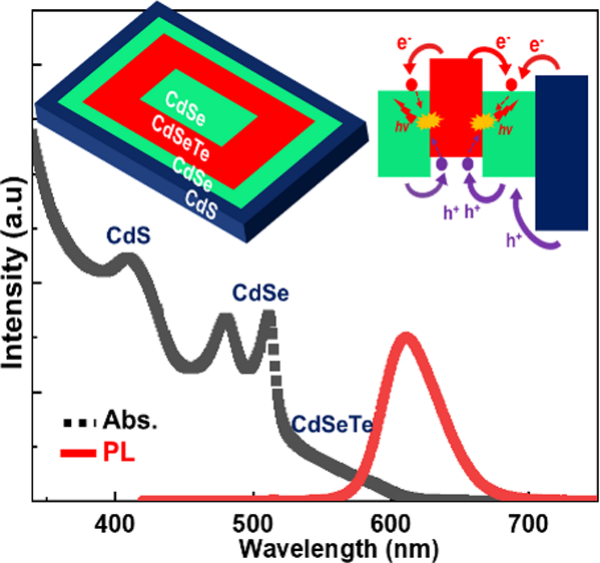

Solution-processed two-dimensional nanoplatelets (NPLs) allowing lateral growth of a shell (crown) by not affecting the pure confinement in the vertical direction provide unprecedented opportunities for designing heterostructures for light-emitting and -harvesting applications. Here, we present a pathway for designing and synthesizing colloidal type-II core/(multi-)crown hetero-NPLs and investigate their optical properties. Stoke’s shifted broad photoluminescence (PL) emission and long PL lifetime (∼few 100 ns) together with our wavefunction calculations confirm the type-II electronic structure in the synthesized CdS/CdSe_1–*x*_Te_*x*_ core/crown hetero-NPLs. In addition, we experimentally obtained the band-offsets between CdS, CdTe, and CdSe in these NPLs. These results helped us designing hetero-NPLs with near-unity PL quantum yield in the CdSe/CdSe_1–*x*_Te_*x*_/CdSe/CdS core/multicrown architecture. These core/multicrown hetero-NPLs have two type-II interfaces unlike traditional type-II NPLs having only one and possess a CdS ending layer for passivation and efficient suppression of stacking required for optoelectronic applications. The light-emitting diode (LED) obtained using multicrown hetero-NPLs exhibits a maximum luminance of 36,612 cd/m^2^ and external quantum efficiency of 9.3%, which outcompetes the previous best results from type-II NPL-based LEDs. These findings may enable designs of future advanced heterostructures of NPLs which are anticipated to show desirable results, especially for LED and lasing platforms.

## Introduction

Solution-processed two-dimensional (2D) nanoplatelets (NPLs) are among the most favorable materials for optoelectronic applications owing to their exceptional optical and electronic characteristics, such as giant oscillator strength,^1^ near-unity quantum yield (QY),^2−4^ large linear and nonlinear absorption cross-sections,^5−7^ exceptionally narrow photoluminescence (PL) emission owing to their well-defined thickness,^1,8,9^ directional emission as a result of in-plane transition dipoles,^10,11^ switchable excitonic polarization,^12^ and low threshold optical gain both in solution and film.^7,13,14^ These 2D semiconductor materials also provide an excellent ground for designing advanced heterostructures because of the ability to grow a shell as well as a crown (laterally grown shell without changing the thickness), which is not accessible for colloidal quantum dots (QDs).^15−17^ Such crown growth provides unique opportunities for designing heterostructures of these NPLs without changing the confinement in each domain.

Recently, heterostructures of NPLs having different electronic and optical properties have been obtained with the growth of a crown on seed CdSe NPLs.^17−19^ CdSe/CdS core/crown NPLs with a type-I electronic structure^9,20^ and CdSe/CdTe core/crown NPLs with a type-II electronic structure have been investigated and demonstrated by several research groups.^21−24^ Since wavefunctions of electrons and holes are localized in spatially separated domains in type-II heterostructures, the reduced oscillator strength leads to longer PL lifetimes compared to type-I heterostructures. Hence, the PL lifetime of CdSe/CdTe NPLs is two orders of magnitude longer than those of the CdSe core and CdSe/CdS core/crown NPLs having a type-I electronic structure.^11,20−22^ In addition, CdSe/CdSe_1–*x*_Te_*x*_ core/alloyed-crown hetero-NPLs have been successfully synthesized with controlled alloying in the crown.^25−27^ The alloying in these type-II hetero-NPLs provides a tuning knob for controlling the electronic and optical properties,^28^ which is significantly crucial for enhancing the performance of hetero-NPLs in light-harvesting and -emitting applications. These designed hetero-NPLs having an alloyed CdSe_1–*x*_Te_*x*_ crown exhibit superior optical properties, such as improved PLQY and slower Auger recombination compared to hetero-NPLs with a pristine CdTe crown.^25−27^

Most recently, the growth of multiple crowns has also been demonstrated on seed CdSe NPLs.^15,16,29,30^ CdSe/CdTe/CdSe double-crowned NPLs were used to demonstrate bicolor power-tunable emission and LEDs with a high external quantum efficiency (EQE).^29,30^ CdSe/CdSe_1–*x*_Te_*x*_/CdS core/multi-crown NPLs were shown to exhibit ultralow amplified spontaneous emission and enhanced photostability as well as reduced stacking which is significantly important for attaining uniform film formation.^15^ In addition, multiexcitonic emission and two-photon fluorescence upconversion were demonstrated using CdSe/CdS/CdTe core/multicrown NPLs.^16^ Although a CdS crown was used as a barrier layer between the CdSe core and the CdTe end region in the work of Khan et al., the band offsets suggest that the electronic structure between CdS and CdTe is type-II and the possibility of recombination at the interface between CdS and CdTe was neglected in this work.^16^ The investigation of optical and electronic properties of CdS/CdTe core/crown NPLs is unambiguously needed to understand the electronic structure and recombination channels in such multicrown hetero-NPLs. In addition, such growth of CdTe on CdS NPLs will reveal the previously unknown band offsets between CdS and CdTe in NPLs. By combining this knowledge with the already known band offsets between CdSe and CdTe, it would also be possible to obtain the band offsets between CdS and CdSe domains in these highly confined 2D semiconductors. Such knowledge of the band offsets is highly valuable for designing hetero-NPLs in different geometries, such as core/crown, core/multicrown, core/shell, and core/crown/shell. Hence, the characterization of the electronic and optical properties of CdS/CdTe NPLs is crucial for understanding the optical properties and designing hetero-NPLs, which unavoidably employ CdS and/or CdTe layers as either intermediate or final layers on the seed CdSe NPLs.

In this study, we present the synthesis and optical characterization of CdS/CdSe_1–*x*_Te_*x*_ core/crown hetero-NPLs exhibiting a type-II electronic structure. An atomically flat 4 monolayer (ML) CdSe_1–*x*_Te_*x*_ crown was grown on the seed 4 ML CdS NPLs, and the optical properties of these type-II NPLs were systematically studied by varying the alloying level in the crown. In these CdS/CdSe_1–*x*_Te_*x*_ core/crown hetero-NPLs, the PL emission peak is Stoke’s shifted and the PL lifetime is in the order of hundreds of nanoseconds, which is two orders of magnitude longer than that of CdS core NPLs. These results signify type-II band alignment in these CdS/CdTe core/crown hetero-NPLs together with our wavefunction calculations. In addition, the band offsets between CdS and CdTe domains were experimentally obtained through PL and absorption measurements. The experimentally acquired conduction band (CB) offset is 0.16 eV, and the valence band (VB) offset is 0.97 eV for the CdS/CdTe core/crown NPLs. The found offsets between CdS and CdTe helped us to obtain the offsets between the CdS and CdSe for these NPLs, as the offsets between CdSe and CdTe are known for NPLs. As a result, we obtained the offsets between CdS, CdTe, and CdSe, which are crucial for designing advanced heterostructures of NPLs.

These results allowed us to design functional multicrown hetero-NPLs with a high PLQY of 97% in the CdSe/CdSe_0.7_Te_0.3_/CdSe/CdS core/multicrown architecture. Our designed multicrown hetero-NPLs with their two type-II interfaces are superior to the previously reported type-II core/crown and core/multicrown NPLs having only one type-II interface. In addition, the CdS crown in our designed hetero-NPLs ensures the effective passivation of the periphery and inhibition of stacking, which are required for obtaining smooth films with negligible roughness. The light-emitting diode (LED) obtained using these CdSe/CdSe_0.7_Te_0.3_/CdSe/CdS core/multicrown hetero-NPLs exhibits a maximum EQE of ∼9.3%, which surpasses the previously reported best EQE from type-II NPL-based LEDs. This LED emitting at 624 nm exhibits colors well located in the red region with (*x*, *y*) chromaticity coordinates of (0.63, 0.36), which are highly desired for achieving high performance in the red region due to the significant further decay of the eye sensitivity curve at the longer wavelengths in the deep-red region.^31−33^

## Results and Discussion

**Figure 1:**
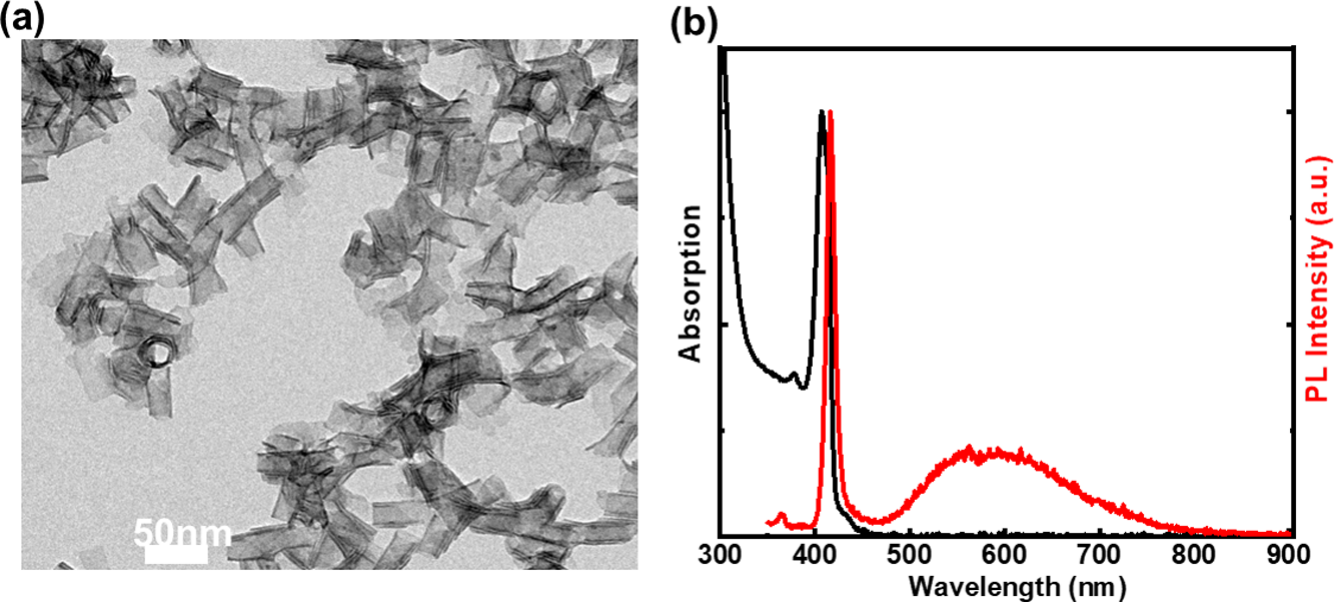
(a) TEM image of 4 ML CdS NPLs. (b) Absorption and PL spectra of 4 ML thick CdS NPLs.

The core/(multi)crown type-II NPLs were synthesized via a seed mediated growth method. To acquire CdS/CdSe_1–*x*_Te_*x*_ core/crown NPLs, first, 4 ML CdS seed NPLs were synthesized by employing an acetate salt to facilitate lateral growth, as reported previously.^9,34^ The obtained seed CdS NPLs are rolled up to generate a tubular form with an axial length of 55 nm ± 10 nm as can be seen from the transmission electron microscopy (TEM) image in Figure 1a. The PL and absorption spectra of the 4 ML CdS seed NPLs are given in Figure 1b. The absorption peak around 406 nm is associated with the merged heavy hole and light hole transitions in 4 ML CdS NPLs.^1,9^ The narrow PL peak appearing at 421 nm with a full-width-at-half-maximum (FWHM) of 12 nm is associated with the heavy hole transition, while the broad emission occurring at 645 nm is due to the deep trap emission.

**Figure 2:**
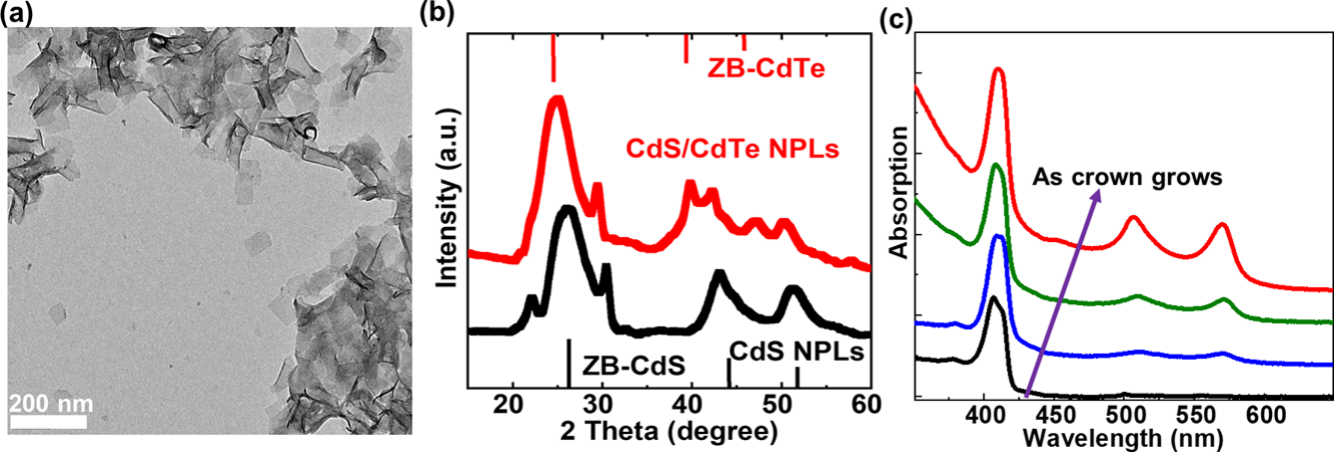
(a) Representative TEM image of 4 ML CdS/CdTe core/crown NPLs. (b) XRD spectrum of 4 ML CdS (black) and CdS/CdTe (red) core/crown NPLs. (c) Evolution of the absorption spectrum of CdS/CdTe core/crown NPLs as CdTe crown grows.

CdS/CdSe_1–*x*_Te_*x*_ core/crown NPLs were obtained by using the CdS NPLs as seeds and the growth of CdTe crown was accomplished by introducing cadmium acetate homogenously dispersed in a mixture of oleic acid and octadecene (ODE) at room temperature and then continuously injecting SeTe precursor (ODE-trioctylphosphine-Se_1–*y*_Te_*y*_) at 200 °C.^21^ The injection amount of the SeTe precursor was adjusted to monitor the lateral size of the crown. The composition of the 0.03 M ODE-TOP-Se_1–*y*_Te_*y*_ mixture can be adjusted by changing the relative amounts of added ODE-trioctylphosphine-Se and ODE-trioctylphosphine-Te to control the composition in the resulting CdSe_1–*x*_Te_*x*_ crown. For example, to obtain CdS/CdTe core/crown NPLs, we only injected 0.03 M ODE-TOP-Te solution at a rate of 15 mL/h. Further details of the crown growth synthesis are provided in the Supporting Information (SI). A TEM image of the resulting CdS/CdTe core/crown NPLs is shown in Figure 2a. CdS/CdTe core/crown NPLs similar to seed CdS NPLs are rolled up into a tubular form and their axial length is increased to 92 ± 15 nm, which confirms the lateral growth of the CdTe crown on CdS NPLs. Additionally, X-ray diffraction (XRD) patterns of the CdS core and CdS/CdTe core/crown NPLs exhibiting a zinc-blend crystal structure are presented in Figure 2b. In the XRD spectrum of CdS NPLs, only the XRD peaks associated with the zinc blende CdS appear as presented in Figure 2b. However, the growth of the CdTe crown on CdS core NPLs results in the emergence of additional new peaks related to the zinc blende CdTe lattice and a slight shift in the angles of the peaks associated with the CdS phase. This slight shift toward smaller angles in the CdS lattice is due to the contraction of the CdSe lattice as a result of the built-up strain due to the lattice mismatch and a larger lattice constant of the CdTe crown.

The evolution of the absorption spectrum of the CdS/CdTe NPLs as the Te precursor injected continuously is given in Figure 2c. A narrow peak at 570 nm in the absorption spectra emerges after starting the injection of the Te precursor into the solution having seed CdS NPLs. The increase in the intensity of this peak at 570 nm is associated with the growth of the CdTe crown, while the absorption peak around 410 nm is associated with the heavy and light hole transitions taking place in the 4 ML CdS core.^1,16^ In these CdS/CdTe core/crown NPLs, the spectral position of excitonic features associated with the CdS core remains almost unchanged with the CdTe crown growth since the growth is only in the lateral directions. The peak around 570 nm in the absorption spectra is related to the heavy hole excitonic transition taking place in the CdTe crown.^16^ For 4 ML CdTe NPLs, the heavy hole transition occurs at 556 nm as reported earlier.^21^ This redshift observed in the heavy hole transition of CdTe in our core/crown heterostructure and previously reported core/multicrown hetero-NPLs^16^ compared to that of core CdTe NPLs can be explained by the leakage of the exciton wavefunction into the CdS core and the change in the dielectric compared to core CdTe NPLs. Similarly, a redshift in heavy hole excitonic transitions of CdSe in CdSe/CdS and CdS/CdSe core/crown NPLs was observed compared to heavy hole excitonic transitions of the core only CdSe NPLs.^9,20^ In addition, this heavy hole transition at 570 nm is another absolute signature of successful growth of CdTe crown on CdS NPLs, since if there is growth of separate CdTe NPLs, we should observe the heavy hole transition at ∼556 nm. The PL peak of the CdS/CdTe core/crown NPLs is located at 613 nm and strongly redshifted compared to the band-edge PL emission peaks of 4 ML CdS and CdTe NPLs.^1^ This redshifted emission is a key signature of the recombination of spatially indirect excitons at the core/crown interface similar to the redshifted PL emission previously reported from type-II CdSe/CdTe core/crown NPLs.^21−23^

**Figure 3:**
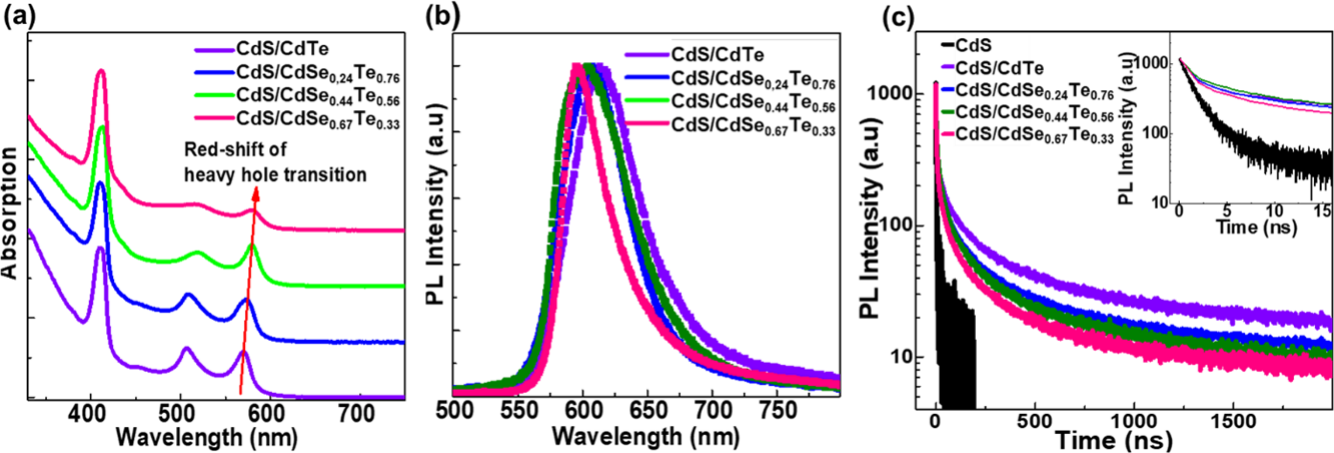
(a) Absorption spectra of CdS/CdSe_1–*x*_Te_*x*_ core/crown NPLs. (b) PL spectra of CdS/CdSe_*x*_Te_1–*x*_ core/crown NPLs. (c) TRPL decay curves of CdS core and CdS/CdSe_1–*x*_Te_*x*_ core/crown NPLs. The inset of (c) shows the TRPL measurements on a shorter temporal window to make the initial decays visible.

The absorption and PL spectra of CdS/CdSe_1–*x*_Te_*x*_ core/crown NPLs having different alloying levels in the crown are presented in Figure 3a,b, respectively. The excitonic features can be modified and tuned by changing the crown composition. The energy of heavy hole transition taking place in the CdSe_1–*x*_Te_*x*_ crown shifts from 570 to 583 nm as the Se concentration is increased as presented in Figure 3a. In addition, in all CdS/CdSe_1–*x*_Te_*x*_ core/crown NPLs, the PL peak is redshifted compared to the heavy hole transitions of CdS and CdSe_1–*x*_Te_*x*_ suggesting still effective type-II band alignment. As shown in Figure 3b, PL redshifts continuously as the Te concentration in the crown is increased. This continuous redshift in the PL peak can be explained by the increase in the VB offset between CdS and CdSe_1–*x*_Te_*x*_ as the Te concentration is increased as tabulated in Table 1. Thus, because of this increase in the VB offset with the increase of *x* for *x* > 0.33, the energy of the PL peak is continuously redshifting as a result of the recombination of the holes confined in the VB of the CdSe_1–*x*_Te_*x*_ crown and electrons localized in the CB of the CdS core. Therefore, in other words, while the blueshifts in the absorption spectra can be attributed mainly to the strong increase in the CB offset (from 0.06 to 0.16 eV in CdS/CdSe_1–*x*_Te_*x*_ core/crown NPLs as presented in Table 1) induced by the additional Te incorporation into the crown (as *x* increasing for *x* > 0.33), the redshifting of the PL by the additional Te incorporation into the crown (for *x* > 0.33) is due to the increase in the VB offset. This strong increase in the CB offset (from 0.06 to 0.16 eV) leading to stronger localization of electrons in the CdS crown, as the Te concentration is increased from *x* = 0.33 to *x* = 1 in the CdSe_1–*x*_Te_*x*_ alloyed crown, can also be followed by the PL lifetime measurements, which are presented in the following section.

**Table 1:** CB and VB Offsets of CdS/CdSe_1–*x*_Te_*x*_ Core/Crown NPLs Obtained from the Absorption and PL Measurements

CdS/CdSe_1–*x*_Te_*x*_ core/crown NPLs		
atomic percentage of Te/(Se + Te) (%)	CB offset (eV)	VB offset (eV)
100	0.16	0.97
76	0.11	0.94
56	0.08	0.93
33	0.06	0.92

To further explore the carrier recombination dynamics and type of band alignment, we performed time-resolved PL (TRPL) spectroscopy to investigate the fluorescence emission decay on CdS/CdSe_1–*x*_Te_*x*_ core/crown NPLs at room temperature. TRPL measurements were carried out on CdS/CdSe_1–*x*_Te_*x*_ core/crown NPLs dispersed in hexane under low excitation power (⟨*N*⟩ < < 0.1) using a pulsed picosecond laser at 375 nm. The PL decay curves obtained at the peak emission wavelength of each set of CdS/CdSe_1–*x*_Te_*x*_ core/crown NPLs are presented in Figure 3c and they were fitted to a three-exponential decay function. Results of the fittings (lifetimes, coefficient of each lifetime, and intensity averaged lifetimes) are summarized in Table S2. The intensity averaged lifetime of CdS/CdTe core/crown NPLs is ∼653.8 ns, similar to the previously reported PL lifetimes of CdSe/CdTe core/crown NPLs and two orders of magnitude longer than the PL lifetime of pristine CdS core NPLs and core/shell NPLs.^21,22,35^ This long PL lifetime compared to the pristine CdS NPLs suggests the presence of type-II band alignment in this system where holes are localized around the crown and electrons are confined in the core. Although the PL lifetime from CdS/CdSe_1–*x*_Te_*x*_ core/crown NPLs is shorter than that of CdS/CdTe core/crown NPLs, it is still two orders of magnitude longer than that of CdS NPLs as summarized in Table S2. The PL lifetime of CdS/CdSe_1–*x*_Te_*x*_ core/crown NPLs is shortened as the Te concentration is decreased as presented in Figure 3c due to the strong decrease in the CB offset (from 0.16 eV for *x* = 1 to only 0.06 eV for *x* = 0.33 in CdS/CdSe_1–*x*_Te_*x*_ core/crown NPLs as presented in Table 1), suggesting a transition from type-II to a quasi-type-II carrier localization regime. This decrease in the lifetime as the Se concentration is increased is mainly because of the increase in the wavefunction overlap of the photogenerated carriers, which results in an increase in the oscillator strength, hence promoting the radiative recombination rate.

**Figure 4:**
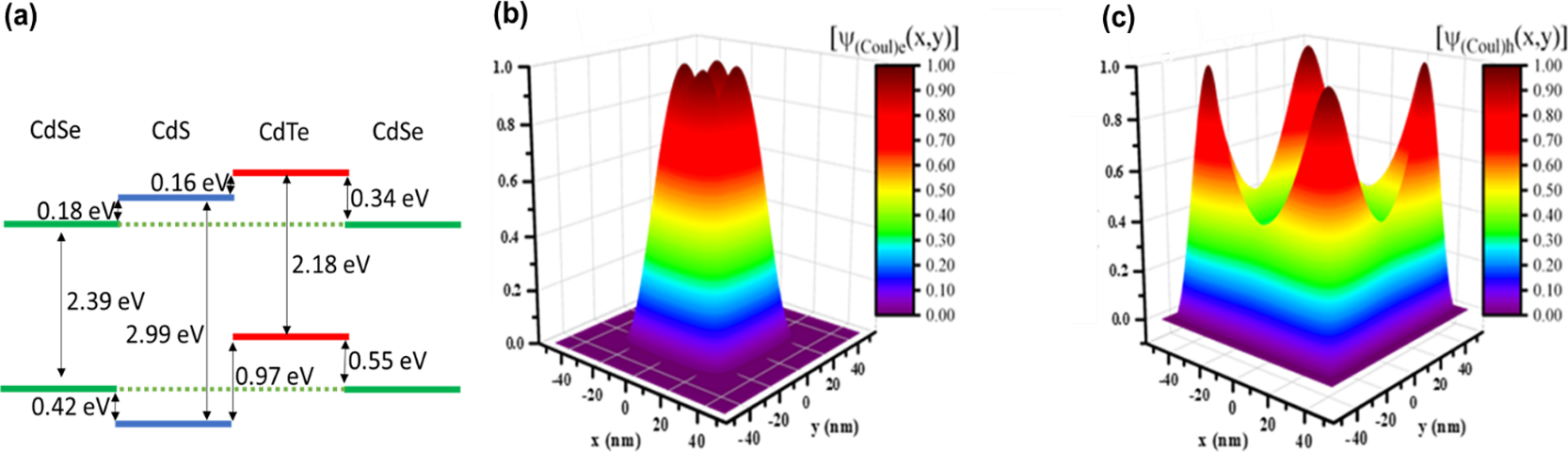
(a) Obtained band offsets between 4 ML CdS, CdSe, and CdTe NPLs. Calculated distribution of (b) electron and (c) hole wavefunctions in CdS/CdTe core/crown NPLs with Coulomb interactions included. As shown in (b) electrons are strongly localized in the CdS core, while holes are strongly localized in the CdTe crown as presented in (c) due to the band offsets and large lateral area of the NPLs. The attractive Coulomb interactions between the electron and hole lead to slightly stronger localization of the electron wavefunction on the corners (compared to the results for the free particle case (no Coulomb Interactions) presented in Figure S7) while the hole wavefunction is not modified visibly with the introduction of the Coulomb interactions.

To further prove the generation of a type-II junction in the CdS/CdTe core/crown hetero-NPLs, we used the spectral position of the peaks associated with the heavy hole transitions of CdS and CdTe domains and the spectral position of the emission from the CdS/CdTe hetero-NPLs. The PL emission at 2.02 eV as a result of the indirect transition between spatially separated electrons localized in the CdS core and holes confined in the CdTe crown of the heterojunction is strongly redshifted compared to the band edge transitions taking place in the CdS core and CdTe crown located at 2.98 and 2.17 eV, respectively. Hence, the energy of the PL peak from the type-II transition is determined by the energy of the heavy hole transitions taking place in the CdS core and CdTe crown and their relative band offsets.^23^ Using the transition energies observed in the absorption spectrum and the PL peak energy, we obtained the CB offset of 0.16 eV and VB offset of 0.97 eV in the CdS/CdTe core/crown hetero-NPLs as shown in Figure 4a. These obtained band offsets signify the formation of a type-II band alignment in CdS/CdTe NPLs along with the PL lifetime results. These band offsets are slightly different from the bulk band offsets of the bulk CdS/CdTe, which can be attributed to the formation of lattice strain and confinement in these hetero-NPLs as the confinement effects can significantly change the band offsets and type of the junction as previously shown in CdTe/CdS core/shell QDs.^36,37^

In addition, we synthesized CdSe/CdTe core/crown NPLs to obtain the band offsets between CdSe, CdS, and CdTe in 4ML hetero-NPLs in core/(multi)-crown architecture having CdSe, CdS, and CdTe as the core or crown. The obtained band offsets are significantly important since 4 ML NPLs are used as a model system in most of the studies by the community of the NPLs. The PL and absorption spectra of CdSe/CdTe core/crown NPLs are provided in Figure S2. By using the spectral position of the peaks associated with the heavy hole transitions of CdSe and CdTe regions and the spectral position of the emission from the CdSe/CdTe hetero-NPLs, we obtained the band offsets between the 4ML CdSe core and CdTe crown. Combining these offsets with the offsets between CdS and CdTe enables us to obtain the band offsets of CdS, CdTe, and CdSe as shown in Figure 4a. As presented in Figure 4a, while CdS/CdTe and CdSe/CdTe core/crown NPLs exhibit a type-II electronic structure, CdSe/CdS NPLs show type-I band alignment with a CB offset of 0.18 eV and a VB offset of 0.42 eV.

After obtaining the band offsets of the CdS/CdTe core/crown NPLs, we calculated the wavefunction distribution of the photogenerated holes and electrons in CdS/CdTe core/crown hetero-NPLs to explore the electronic structure. Wavefunctions and probability distributions were obtained by solving the problem of a particle-in-a-box in two dimensions taking into account the lateral size of the hetero-NPLs and using the stationary Schrödinger equation with effective mass approximation. In addition, the electron–hole Coulomb interactions were introduced using the perturbation theory. Important parameters of the calculations are the band offsets, obtained experimentally as given in Figure 4a, the lateral sizes of the CdS core and CdTe crown region which are obtained using the TEM images, and effective masses which were obtained from the previous reports.^1^ 2D probability distribution maps of electrons and holes in the lateral area of these hetero-NPLs are given in Figure 4b,c, respectively. As can be seen in Figure 4, electrons are strongly confined in the CdS core due to the existing potential barrier and large lateral size of the core, while holes are strongly localized in the CdTe crown due to the large VB offset in the range of 1 eV and a larger effective mass of holes. It is worth noting that the attractive Coulomb interactions between the electron and the hole lead to stronger localization of the electron wavefunction on the corners of the CdS core (compared to the results for the free particle case (i.e., the case of *no Coulomb interactions*) presented in Figure S7), while the hole wavefunction is not modified visibly with the introduction of the Coulomb interactions. The large lateral size of the CdS core is essential to keep the electrons localized in the core since the band offset is not very strong. For example, in the case of CdSe/CdS core/shell NPLs having a few nanometer thick core and shell with a small CB offset in the order of ∼100 meV and in the case of CdS/CdTe QDs having a few MLs of CdTe, the electrons are distributed mainly in the whole structure.^11,37,38^ However, the large lateral area of the CdS core plays a critical role in keeping electrons residing only in the core, although the band offset is not significantly strong. Hence, this large area of the core leads to the formation of a type-II electronic structure rather than a quasi-type-II structure in these CdS/CdTe core/crown hetero-NPLs, which is also supported by the long PL lifetimes in the order of hundreds of nanoseconds and Stoke’s shifted PL emission.

In addition, we calculated the wavefunctions and probability distributions of photogenerated carriers of 4 ML CdTe/CdS core/crown NPLs having core and crown sizes similar to our CdS/CdTe core/crown NPLs using the same approach described above as presented in Figure S8. Similar to CdS/CdTe NPLs, we observed that the photogenerated carriers are spatially separated in these CdTe/CdS core/crown NPLs and hence they exhibit a type-II electronic structure. However, we expect a faster transition to a quasi-type-II electronic structure in these CdTe/CdS core/crown NPLs compared to CdS/CdTe core/crown NPLs as the lateral size of the crown is reduced due to faster leakage of electron wavefunction to the CdTe core as a result of the weak CB offset and smaller effective mass of electrons compared to those of holes.

After confirming that CdS/CdTe core/crown NPLs exhibit a type-II electronic structure, we explored a new type of type-II hetero-NPL combining the type-II electronic structure with a wide gap semiconductor for passivation of the sites on the periphery for high efficiency light emitting applications, such as LEDs and lasing. Previously synthesized CdSe/CdSe_1–*x*_Te_*x*_ core/crown NPLs with their high PLQYs (slightly above ∼90%)^25^ present an excellent platform for further design of complex type-II NPL architectures. Unfortunately, these CdSe/CdSe_1–*x*_Te_*x*_ core/crown NPLs are prone to strong stacking, which limits their usage in optoelectronic applications, and lack a wide gap semiconductor with suitable band offsets to keep the electron and hole away from the periphery to avoid nonradiative trap sites on the periphery.^15^ Adding a CdS layer on these type-II NPLs was shown to strongly reduce the stacking phenomenon.^15^ However, these previously developed CdSe/CdTe/CdS core/double-crown NPLs are still not best fit for desired applications mainly due to the type-II band alignment between CdTe and CdS crown layers as shown in this present work and the significant lattice mismatch of ∼10% between CdTe and CdS which leads to the formation of nonradiative defect sites and growth of CdS crown almost only on the corners.^15^ Such a formation of defect sites, which promotes nonradiative recombination, is detrimental for light emitting applications, e.g., LEDs and luminescent solar concentrators. Adding a CdSe crown layer between CdSeTe and CdS crowns is expected to ease the lattice mismatch between the CdSe_1–*x*_Te_*x*_ and CdS crown layers, which in turn assists uniform growth of CdS layer on these CdSe/CdSe_1–*x*_Te_*x*_/CdSe core/multicrown hetero-NPLs and inhibit an additional undesirable type-II recombination pathway due to the type-II band alignment between CdTe and CdS crown layers. The lattice mismatch between CdSe and CdS is only 5%, much lower compared to that of CdSe and CdS. Hence, the addition of a CdSe crown layer between the CdSe_1–*x*_Te_*x*_ and CdS layers mediates the lattice mismatch and strain. This proposed CdSe/CdSe_1–*x*_Te_*x*_/CdSe/CdS core/multicrown architecture is expected to be superior to previously generated type-II core/multicrown hetero-NPLs with its two type-II interfaces (between CdSe core and CdSe_1–*x*_Te_*x*_ crown and between CdSe_1–*x*_Te_*x*_ crown and CdSe crown), CdSe buffer layer to compensate for the lattice mismatch and strain and CdS crown layer acting as a wide bandgap periphery passivator and stacking inhibitor.

**Figure 5:**
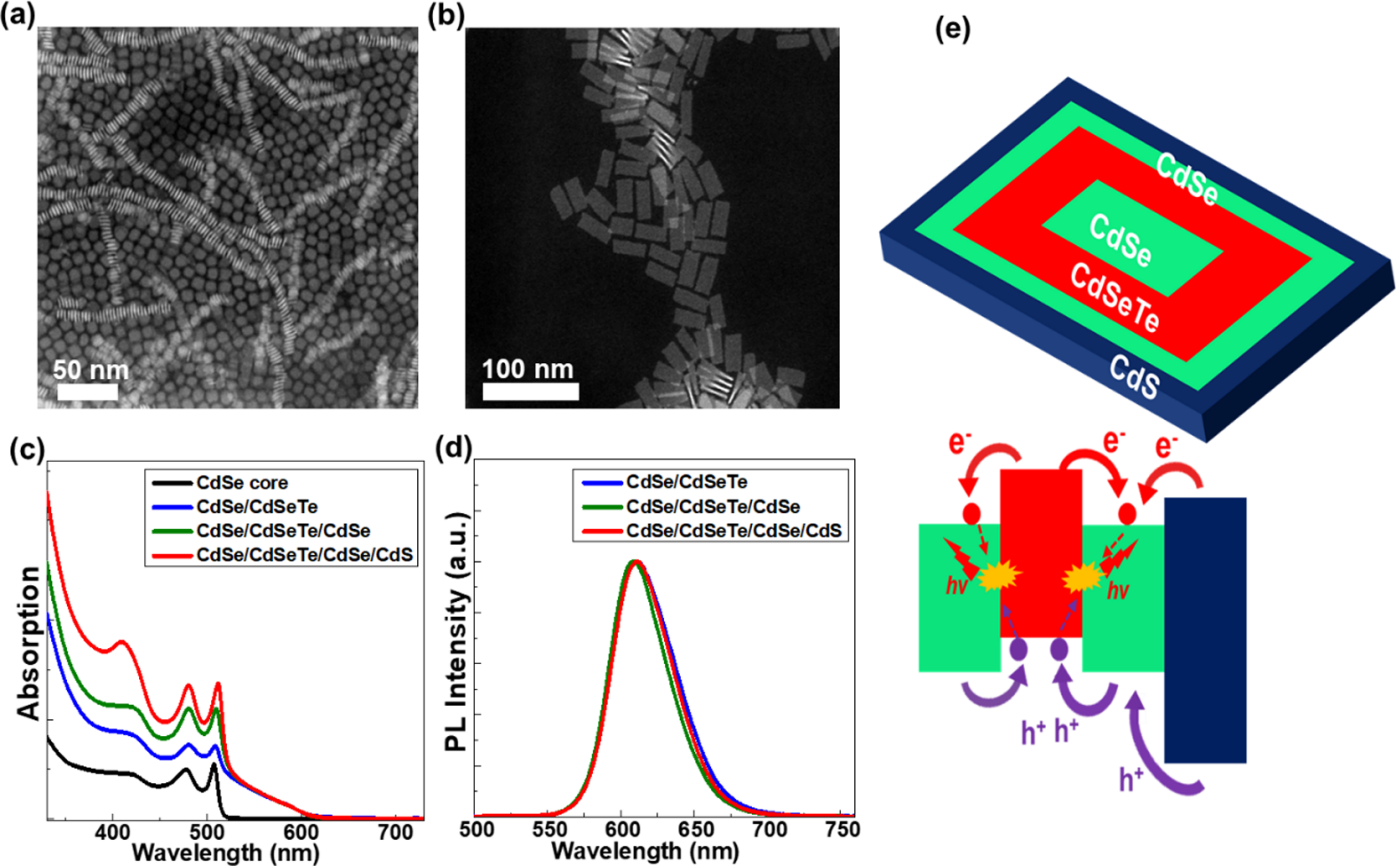
Representative TEM images of (a) seed 4 ML CdSe NPLs and (b) CdSe/CdSe_0.7_Te_0.3_/CdSe/CdS core/multicrown hetero-NPLs. (c) Absorption spectra of the CdSe core, CdSe/CdSe_0.7_Te_0.3_ core/crown, CdSe/CdSe_0.7_Te_0.3_/CdSe core/multicrown, and CdSe/CdSe_0.7_Te_0.3_/CdSe/CdS core/multicrown NPLs. The absorption spectra of the NPLs having the CdSe_0.7_Te_0.3_ crown are normalized at 550 nm. The absorption spectra were obtained during the subsequent growth of crown layers, and the spectral positions of the emerging peaks signify the successful growth of each crown layer. (d) PL spectra of the synthesized CdSe/CdSe_0.7_Te_0.3_ core/crown, CdSe/CdSe_0.7_Te_0.3_/CdSe core/multicrown, CdSe/CdSe_0.7_Te_0.3_/CdSe/CdS core/multicrown NPLs, which were obtained during the subsequent growth of crown layers. (e) Schematics of CdSe/CdSe_0.7_Te_0.3_CdSe/CdS core/multicrown NPLs and recombination pathways of photogenerated carriers in these core/multicrown NPLs.

We synthesized CdSe/CdSe_1–*x*_Te_*x*_/CdSe/CdS core/multicrown NPLs using seed-mediated growth. First, 4 ML CdSe NPLs were grown to be used as seeds. Subsequently, CdSe_0.7_Te_0.3,_ CdSe, and CdS crowns were grown on the seeds. The size of each crown layer was adjusted by controlling the amount of the injected precursors. Details of the synthesis are provided in the SI. The TEM images of the seed 4ML CdSe NPLs and CdSe/CdSe_1–*x*_Te_*x*_/CdSe/CdS core/multicrown NPLs are shown in Figure 5a,b, respectively. The seed 4 ML CdSe NPLs almost have a square shape with a lateral size of 7 nm × 7 nm while CdSe/CdSe_0.7_Te_0.3_/CdSe/CdS core/multicrown NPLs have a rectangular shape and their lateral size is 15 nm × 36 nm. The variation in the lateral size of the CdSe/CdSe_0.7_Te_0.3_/CdSe/CdS core/multicrown NPLs is small, as can be seen in the TEM images, which indicates uniform growth of crown layers, unlike the CdSe/CdSe_1–*x*_Te_*x*_/CdS core/multicrown NPLs reported previously having a CdS crown almost only around the edges.^15^

In addition, we followed the growth of each crown layer spectrally by taking absorption and PL measurements of aliquots at each growth stage, which are shown in Figure 5c,d. The seed 4ML CdSe NPLs exhibit a heavy hole transition at 508 nm and with the growth of the CdSe_0.7_Te_0.3_ crown layer a broad absorption peak beyond 510 nm emerges. This new absorption feature beyond 510 nm signifies the successful growth of the CdSe_0.7_Te_0.3_ layer on the seeds.^25−27^ Moreover, the emergence of a broad PL peak at 611 nm with an FWHM of 49 nm signifies the successful growth of the CdSe_0.7_Te_0.3_ crown layer. The PLQY of these obtained CdSe/CdSe_0.7_Te_0.3_ core/crown NPLs is 85% which is similar to the previously reported PLQY of CdSe/CdSe_1–*x*_Te_*x*_ core/crown NPLs in reference (25). The PLQY measurements were performed using an integrating sphere with 400 nm excitation. The further expansion of the CdSe crown layer can be observed as the intensity of the heavy hole transition of 4ML CdSe located around 510 nm increases in the absorption spectrum. In addition, the PL peak blue-shifts slightly to 609 nm and the FWHM decreases to 46 nm. Moreover, the PLQY of CdSe/CdSe_0.7_Te_0.3_ core/crown NPLs increases from 85 to 94% with the growth of the CdSe crown on CdSe/CdSe_0.7_Te_0.3_ core/crown NPLs. The growth of the final CdS crown layer can be validated by the emergence of a new peak at 410 nm in the absorption spectrum, which corresponds to the excitonic transitions of 4ML CdS. With the growth of the CdS crown layer, the PL peak shifts from 609 to 611 nm and the FWHM becomes 48 nm. These slight changes in the PL spectra as the subsequent crown layers grown after the CdSe_0.7_Te_0.3_ crown can be attributed to the built-up strain and the change in the dielectric. Also, it is worth mentioning that a typical synthesis of core NPLs leads to the formation of NPLs with different thicknesses, which can be clearly observed in the absorption spectra. Therefore, the absence of other peaks belonging to NPLs in various thicknesses in the absorption spectra provided in Figures 5c and 3a rules out the possibility of secondary nucleation of new NPLs during the syntheses of CdSe/CdSe_0.7_Te_0.3_/CdSe/CdS core/multicrown NPLs and CdS/CdSe_1–*x*_Te_*x*_ core/crown NPLs. In addition, we observed a further increase in PLQY, reaching ∼97%, when we grow the final CdS crown. The average PLQY from these CdSe/CdSe_0.7_Te_0.3_/CdSe/CdS core/(multi-)crown NPLs is 95% out of 5 samples produced with a standard deviation of 3%. This small variation is likely due to the small differences in the passivation with the ligands which was affected by the cleaning process. Such an increase in the PLQY with the growth of CdSe and CdS crown layers can be attributed to the improvement in the passivation of the periphery, as similar improvements in PLQY were observed on CdSe NPLs with the growth of the CdS crown layer.^39^ Hence, our CdSe/CdSe_0.7_Te_0.3_/CdSe/CdS core/multicrown NPLs having a double type-II interface and a wide gap passivator with a near unity PLQY present a rational platform for light emitting applications.

The most important parameter to obtain a high QY in these CdSe/CdSe_1–*x*_Te_*x*_/CdS core/multicrown NPLs is the parameter *x* (concentration of Te in the alloyed crown). As given in Figure S3 (in the Supporting Information), the PLQY of CdSe/CdSe_0.3_Te_0.7_/CdSe/CdS core/multicrown NPLs is ∼65%, which is much lower than the PLQY of CdSe/CdSe_0.7_Te_0.3_/CdSe/CdS core/multicrown NPLs. Hence, one can get near unity PLQY (∼97%) with a small variation in the red region, if the composition of the alloyed crown, CdSe_1–*x*_Te_*x*,_ is kept close to *x* = 0.3.

The reproducible high PLQY and the Stoke’s shifted emission make these CdSe/CdSe_0.7_Te_0.3_/CdSe/CdS core/multicrown NPLs excellent candidates for light-emitting applications including LEDs. The recombination pathways showing the origin of PL emission in these CdSe/CdSe_0.7_Te_0.3_/CdSe/CdS core/multicrown having two type-II interfaces are presented in Figure 5e. As shown in the schematic, the CdS crown also helps the further generation of carriers in these hetero-NPLs with photoexcitation and, therefore, it contributes to the absorption cross-section, which is quite useful and important especially for optically pumped lasing applications. The high PLQY achieved under 400 nm excitation in these type-II hetero-NPLs indicates that funneling of the photogenerated carriers from different regions to the interfaces is very efficient.

In these type-II core/multicrown NPLs, it is worth mentioning that the recombination largely takes place in the interface between the CdSe_0.7_Te_0.3_ crown and CdSe crown as a result of the localization of the electrons strongly in the CdSe crown as presented in Figure S9 due to the strong Coulomb interactions. The localization of electrons is strongly affected by the Coulomb interactions, while the localization of the holes is slightly disturbed as can be clearly observed in Figure S9. According to those results, while ∼6% of the electrons are localized in the CdSe core, ∼94% of the electrons are localized in the CdSe crown, and holes are strongly localized only in the CdSe_0.7_Te_0.3_ crown. If we assume near-unity PLQY from both type-II interfaces, ∼6% of the emission originates from the first type-II interface (between the electrons localized in the CdSe core and the holes localized in the CdSe_0.7_Te_0.3_ crown) and ∼94% of the emission comes from the second type-II interface (between the electrons localized in the CdSe crown and the holes localized in the CdSe_0.7_Te_0.3_ crown). Considering the PLQY of 94% from our intermediate structure of CdSe/CdSe_0.7_Te_0.3_/CdSe core/multicrown NPLs and 97% from our final structure, we believe such approximation will likely lead a minor deviation from the actual case.

**Figure 6:**
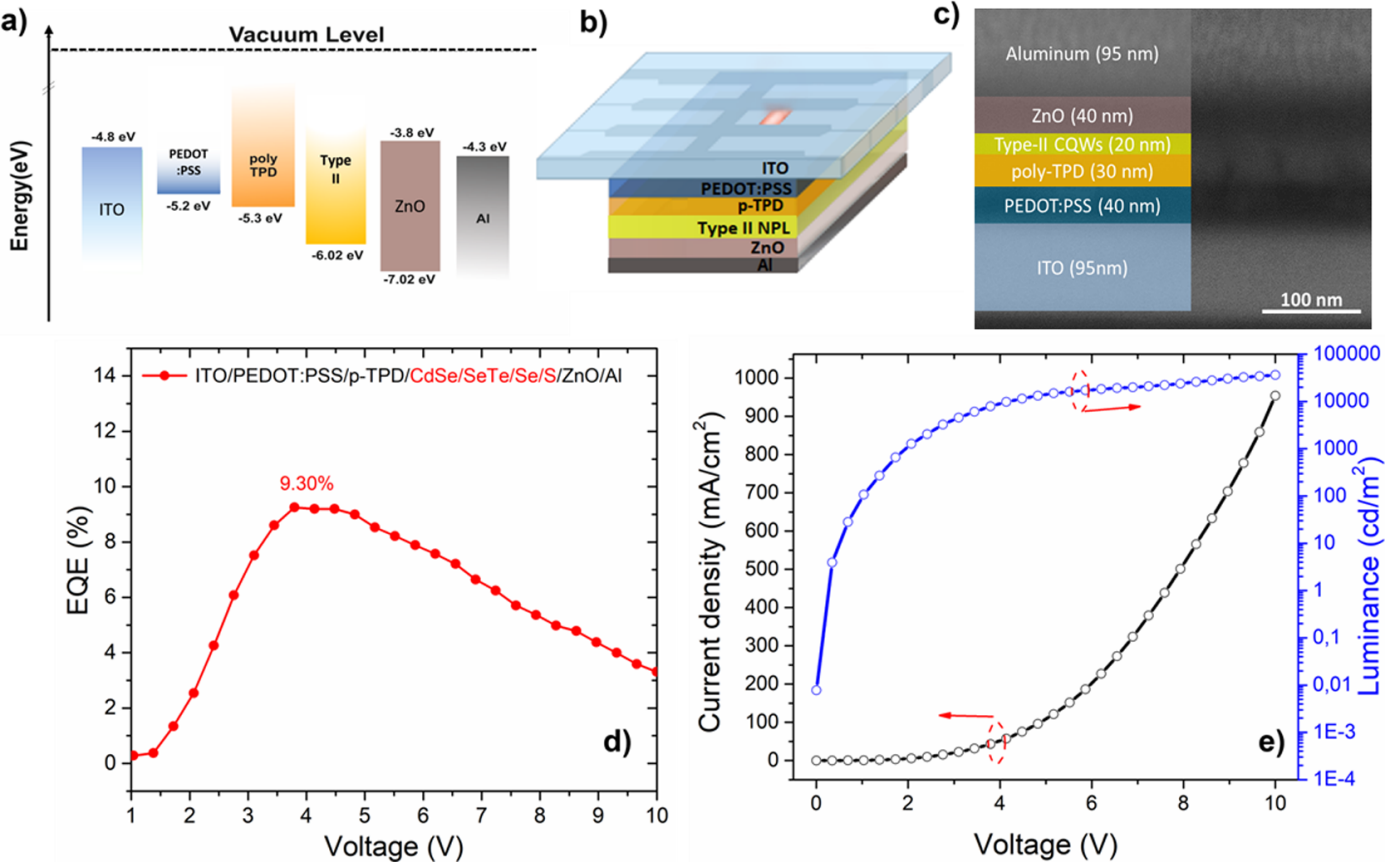
(a) Energy band diagram of the LED structure. (b) Schematic of the device architecture. (c) Cross-sectional FIB image of the LED. (d) Voltage dependence of EQE of our LED. (e) Current density and luminance versus voltage diagrams of our LED.

Motivated by the high PLQY of our CdSe/CdSe_0.7_Te_0.3_/CdSe/CdS core/multicrown NPLs, we fabricated solution-processed LED devices. These designed type-II CdSe/CdSe_1–*x*_Te_*x*_/CdSe/CdS core/multicrown NPLs achieve high PLQY in film owing to reduced reabsorption losses and suppressed energy transfer among neighboring NPLs because of their highly efficient Stoke’s shifted emission.^26^ These features make type-II multicrown hetero-NPLs highly promising candidates as an emissive layer for LEDs with a high EQE. We fabricated LED devices using our type-II CdSe/CdSe_1–*x*_Te_*x*_/CdSe/CdS core/multicrown NPLs to investigate their performance in LEDs. The energy band diagram and device structure of the fabricated LED device based on CdSe/CdSe_0.7_Te_0.3_/CdSe/CdS core/multicrown NPLs are given in Figure 6a,b. The device structure consists of a transparent indium tin oxide (ITO) anode electrode on a glass substrate, a hole injection layer of poly(ethylenedioxythiophene)-polystyrenesulfonate (PEDOT:PSS), a hole transport layer of poly(N,N9-bis(4-butylphenyl)-N,N9-bis-(phenyl)-benzidine) (poly-TPD), a type-II CdSe/CdSe_0.7_Te_0.3_/CdSe/CdS core/multicrown NPL based emitting layer, a ZnO electron transport layer, and an aluminum cathode electrode. A cross-sectional scanning electron microscopy (SEM) image of our conventional LED device is presented in Figure 6c. In this cross-sectional SEM image, all the deposited layers are marked and the thicknesses of all the layers are provided. In this LED structure, poly-TPD was chosen as a hole transport layer due to the lower highest occupied molecular orbital (HOMO) energy level of −5.3 eV^40^ and its high hole mobility favoring hole injection and transport, which helps to increase the recombination rate by confining electrons within the active layer due to the relatively large energy offset at the poly-TPD/type-II NPL interface.

The efficiency of the conventional LEDs is presented in Figure 6d. The EQE of our LEDs reaches 9.3%, which is significantly higher than previous LED devices based on type-II NPLs due to the higher QY of our NPLs and the optimization of each layer in the device.^26^ The device performance shows a slight variation from batch to batch as listed in Table S4. The average maximum EQE from our devices is 7.9% with a standard deviation of 1.0%. In addition, the turn-on voltage of 1.7 V of our LED was acutely low compared to other NPL-based LED devices in the literature.^26,41,42^ At 1000 cd/m^2^, the voltage is still lower than 2 V, which is significantly lower than the previously observed voltage of 3.4 V from type-II CdSe/ CdSe_1–*x*_Te_*x*_ core/crown NPL based LEDs,^26^ and this can be attributed to the efficient charge injection at low bias in our conventional LED. The efficient electron injection in the device facilitated by the well matched high-lying lowest unoccupied molecular orbital (LUMO) levels of Al and ZnO with the LUMO level of CdSe/CdSe_0.7_Te_0.3_/CdSe/CdS NPLs contributes to this reduced turn-on voltage. In addition, for the hole injection, the HOMO energy level of CdSe/CdSe_0.7_Te_0.3_/CdSe/CdS core/multicrown NPLs is compatible with the HOMO energy level of p-TPD; therefore, the hole injection from ITO to the emissive layer is efficient too. Hence, luminance is improved by the efficient recombination of electrons and holes in the emissive layer in these conventional LEDs because of the effective transportation of electrons and holes. The obtained LED device exhibits a maximum luminance of 36,612 cd/m^2^ and a maximum EQE of ∼9%, which outcompetes the previous best results from type-II QDs and NPL-based LEDs. For example, Karatum et al. reported an EQE of 0.53% for the same all solution-processed structure using InP/ZnO.^43^ Lin et al. reported an LED device with a maximum EQE of 6.19% using type-II core/shell QDs as deep-red emitters.^44^ Liu et al. demonstrated an inverted type LED structure with a dual hole transport layer using type-II NPLs which showed a maximum EQE of 3.57% and luminescence of 34,520 cd/m^2^.^26^ Hence, compared to previous type-II QD and NPL-based LEDs, the performance of our devices is remarkably increased.

**Figure 7:**
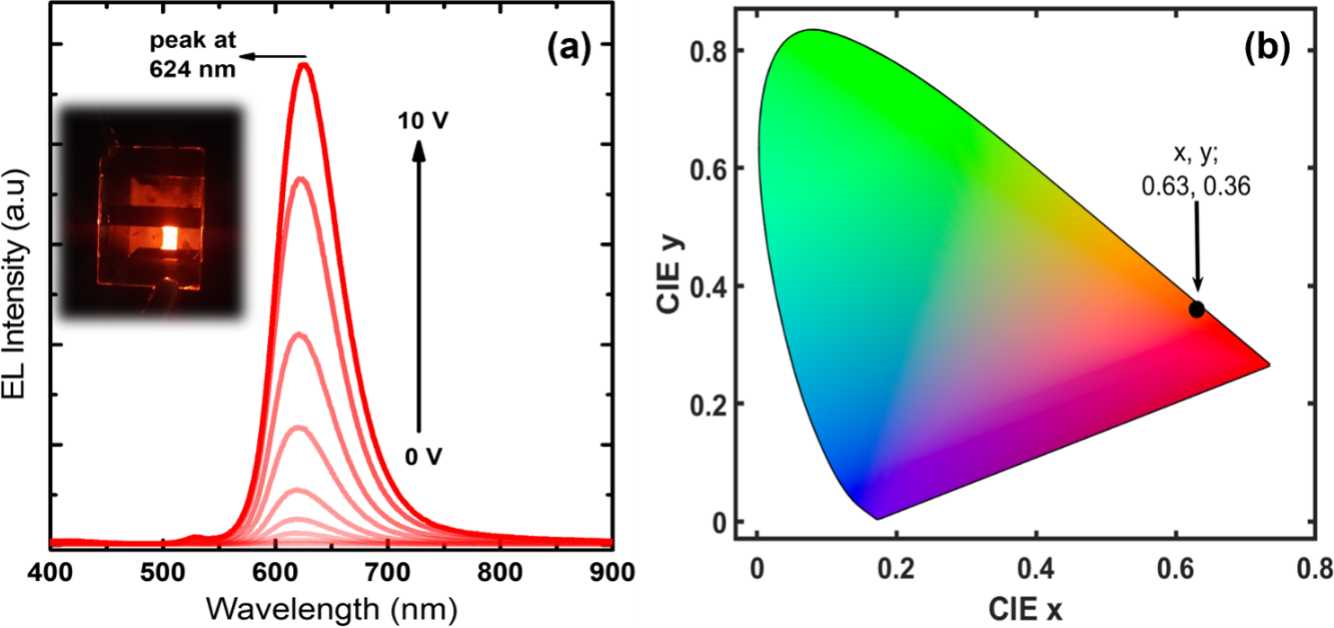
(a) EL spectra of our LED device employing CdSe/CdSe_0.7_Te_0.3_/CdSe/CdS core/multicrown NPLs as an active layer. The inset of (a) shows a photograph of the device operating at 5 V. (b) Corresponding CIE coordinate of our device emitting at 624 nm.

Figure 7a shows the typical electroluminescence (EL) spectrum of the LED device, which exhibits bright EL with an emission peak located at 624 nm with a FWHM of 55 nm at voltages up to 10 V. Our device displays pure color well-located in the red region with (*x*, *y*) tristimulus coordinates of (0.63, 0.36) as presented in Figure 7b. This indicated value is critical for achieving high performance since it provides pure red color at a wavelength where the eye sensitivity is still at a reasonable level. Although one can obtain purer red color from the devices at longer wavelengths, at such wavelengths eye sensitivity drops dramatically as presented in Figure S4, hence limiting the achievement of high-performance devices. For this reason, having the red emission peak wavelength close to 620 nm is critical for achieving high performance and high luminous efficacy of radiation (LER) LEDs.^31−33^

## Conclusions

In conclusion, we demonstrate the synthesis and optical characterization of CdS/CdSe_1–*x*_Te_*x*_ core/crown and CdSe/CdSe_1–*x*_Te_*x*_/CdSe/CdS core/multicrown hetero-NPLs. Long PL lifetime in the order of hundreds of nanoseconds and Stoke’s shifted broad PL emission along with our wavefunction calculations demonstrating the spatial separation of photogenerated carriers indicate the type-II electronic structure in the CdS/CdSe_1–*x*_Te_*x*_ core/crown NPLs. In addition, using the results from our PL and absorption measurements, we experimentally obtained the band offsets between CdS, CdTe, and CdSe, which are critical for constructing advanced structures of NPLs. These findings helped us to build efficient NPLs with a near-unity PLQY in the CdSe/CdSe_1–*x*_Te_*x*_/CdSe/CdS core/multicrown architecture. These multicrown hetero-NPLs include two type-II interfaces plus a CdS crown layer for passivation of the periphery and inhibiting the stacking. This structure is superior compared to previously reported type-II core/multicrown hetero-NPLs because of the inactivation of additional recombination pathways thanks to the separation of the CdSe_1–*x*_Te_*x*_ layer from the CdS layer using CdSe as an intermediate layer. The LED fabricated using these highly efficient type-II core/multicrown hetero-NPLs exhibits a maximum luminance level of 36,612 cd/m^2^ and an EQE of ∼9.3%, which are higher than the previous best results from type-II NPL-based LEDs. In addition, our LED emission wavelength of 624 nm is a desirable parameter for achieving high performance because of the rapidly declining eye sensitivity at longer wavelengths toward the deep red region. These results show the extraordinary potential of advanced heterostructures of type-II NPLs in developing high-performance LEDs. These findings not only present unique optical and electronic properties of type-II multicrown hetero-NPLs but also facilitate the designing of advanced heterostructures of NPLs much needed for light-emitting and -harvesting applications.

## Supporting Information Available

Detailed synthesis procedures of the core and core/(multi-)crown NPLs, results of the fittings from the TRF measurements, absorption and PL spectra of the synthesized core/(multi-)crown NPLs, detailed explanation of wave function distribution calculations in the core/multicrown NPLs and calculated distribution of electron and hole probabilities in the type-II NPLs, eye sensitivity plot, and details of device fabrication and characterization (PDF)
