# Supplementary material for: On the Rational Design of Core/(Multi)-Crown Type-II Heteronanoplatelets
Source: J Am Chem Soc. 2023 May 9;145(22):12033–43. doi: 10.1021/jacs.3c00999 (PMC10251514; doi:10.1021/jacs.3c00999)
Supplement: Supplementary file 1 — ja3c00999_si_001.pdf [file ja3c00999_si_001.pdf]

Supporting Information

On the Rational Design of Core/(multi)-crown Type-II Hetero-Nanoplatelets

Savas Delikanli,[1,2] Betul Canimkurbey,[1,3] Pedro Ludwig Hernández-Martínez,[2] Farzan Shabani,[1] Ahmet Tarik Isik,[1] Ilayda Ozkan,[1] Iklim Bozkaya,[1] Taylan Bozkaya,[1] Furkan Isik,[1] Emek Goksu Durmusoglu,[2] Merve Izmir,[2] Hakan Akgun,[1] and Hilmi Volkan Demir[1,2]*

[1]Department of Electrical and Electronics Engineering, Department of Physics, UNAM – Institute of Materials Science and Nanotechnology, Bilkent University, Ankara 06800, Turkey

[2]Luminous! Center of Excellence for Semiconductor Lighting and Displays, School of Electrical and Electronic Engineering, Division of Physics and Applied Physics, School of Physical and Mathematical Sciences, School of Materials Science and Engineering, Nanyang Technological University, Singapore 639798, Singapore

[3]Serefeddin Health Services Vocational School, Central Research Laboratory, Amasya University, Amasya 05100, Turkey

Chemicals: Sodium myristate, cadmium acetate dihydrate, zinc acetate dihydrate, cadmium nitrate tetrahydrate ($Cd(NO_3)_2.4H_2O$), trioctylphosphine (TOP), sulfur (S), selenium (Se), tellerium (Te), octadecene (ODE), oleic acid (OA), dimethyl sulfoxide (DMSO), tetramethylammonium hydroxide (TMAH, 98%), ethyl acetate, chlorobenzene, ethanol, toluene, hexane, and acetone were purchased from Sigma Aldrich. Poly (N, N′-bis(4-butylphenyl)-N,N′-bis(phenyl)-benzidine) (p-TPD) and poly (ethylene dioxythiophene): polystyrene sulfonate (PEDOT: PSS) were purchased from Lumtec and Osilla, respectively.

Synthesis of cadmium myristate: Cadmium myristate was synthesized following a previously reported protocol with slight modifications.[1] 6.26 g of sodium myristate and 2.46 g of cadmium nitrate tetrahydrate were separately dissolved in 500 and 80 mL of methanol, respectively. Then, the solutions were mixed and stirred vigorously for 5 h with a magnetic stirrer at room temperature. At the end of the reaction, the white precipitate (cadmium myristate) was filtered using a Büchner funnel. The product was washed several times with methanol to remove undesired species and unreacted compounds. Finally, the product was vacuum-dried overnight and stored in ambient conditions.

Synthesis of 4 ML thick CdS NPLs: CdS NPLs with a vertical thickness of 4 ML were synthesized following a method from the literature with slight modifications.[2] First, 217 mg of cadmium acetate dihydrate, 2 mL of 0.1M of ODE-S, 0.24 mL of OA, and 10 mL of ODE were loaded into a three-neck flask. The solution was stirred under an inert atmosphere for 30 min at room temperature. Then, the reaction mixture was heated to 260 °C at the rate of 12 °C/min and kept there for additional 3 min. Then, the reaction was quenched in a water bath. CdS NPLs were precipitated using acetone as an anti-solvent and redispersed in hexane for further use.

Synthesis of 4 ML thick CdSe NPLs: The synthesis of 4 ML CdSe NPLs was carried out using a previously reported recipe.[1] 170 mg of cadmium myristate, 12 mg of Se, and 15 mL of ODE

were loaded into a three-neck flask and degassed under vacuum for an hour at room temperature. Then, the mixture was heated to 240 °C under an inert atmosphere, and as the temperature reached 195°C, 80 mg of cadmium acetate dihydrate was added to the mixture. The reaction was kept at 240 °C for 1 min followed by the injection of 0.5 mL of oleic acid. Finally, the reaction was cooled slowly to room temperature and NPLs were isolated from the side products with selective precipitation using hexane as a solvent and acetone as an anti-solvent.

Preparation of anisotropic growth mixture: 480 mg of cadmium acetate dihydrate, 340 µL of OA, and 2 mL of ODE were loaded into a container and the mixture was sonicated for 30 min at room temperature. Then, the mixture was heated to 160 °C under an ambient atmosphere with continuous stirring. Meanwhile, the mixture was sonicated frequently for 2 min following a 7 min stirring at 160 °C until a white homogeneous viscous compound was obtained.

Synthesis of 4 ML thick $CdS/CdSe_{1-x}Te_x$ core/crown NPLs: $CdS/CdSe_{1-x}Te_x$ core/crown NPLs were prepared by seeded mediated growth method. First, some amount of 4 ML CdS NPLs dispersed in hexane and 15 mL of ODE were loaded into a three-neck flask. The solution was kept under vacuum at room temperature for 1 h to remove hexane from the reaction medium. Then, 0.6 mL of anisotropic growth mixture was injected and the mixture was degassed for 30 min. Next, the temperature was raised to 210 °C under an inert atmosphere and a mixture of 0.03 M TOP−Se and TOP-Te in ODE (0.03 M ODE-TOP-$Se_{1-y}Te_y$), which was prepared in a glovebox under a nitrogen atmosphere, was injected at a rate of 15 mL/h. The composition of 0.03 M ODE-TOP-$Se_{1-y}Te_y$ mixture can be adjusted by changing the relative amounts of added ODE-trioctylphosphine-Se and ODE-trioctylphosphine-Te to control the composition in the resulting $CdSe_{1-x}Te_x$ crown. For example, to obtain CdS/CdTe core/crown NPLs, we only injected 0.03 M ODE-TOP-Te solution at a rate of 15 mL/h. When the desired crown growth was achieved, the reaction was quenched with a water bath and NPLs were isolated with

selective precipitation with hexane as a solvent and ethanol as an anti-solvent. The final product was redispersed and stored in toluene.

Synthesis of 4 ML thick CdSe/CdTe core/crown NPLs: CdSe/CdTe core/crown NPLs were synthesized via a seed mediated growth method. A portion of 4 ML CdSe NPLs synthesis dispersed in hexane and 15 mL of ODE were loaded into a three-neck flask. The solution was kept under vacuum at room temperature for 1 h to remove the hexane from the reaction mixture. Following that, 0.6 mL of anisotropic growth mixture was injected under the nitrogen flow, and then the mixture was degassed for 30 min under vacuum. After this step, the temperature was raised to 210 °C under a nitrogen atmosphere and 0.03M TOP-Te in ODE, which was prepared in a glovebox under a nitrogen atmosphere, was injected at a rate of 15 mL/h. After the injection, the reaction was quenched with a water bath and NPLs were precipitated with the addition of ethanol and hexane. The synthesized core/crown NPLs were dispersed and kept in hexane.

Synthesis of 4 ML thick CdSe/CdSe$_{1-x}$Te$_x$/CdSe/CdS core/multi-crown NPLs: CdSe/CdSe$_{1-x}$Te$_x$/CdSe/CdS core/multi-crown NPLs were synthesized via seed mediated growth method. 4 ML CdSe NPLs dispersed in hexane and 15 mL of ODE were loaded into a three-neck flask. The reaction mixture was degassed at room temperature under vacuum for 1 h to remove hexane from the medium. Then, 2 mL of anisotropic growth mixture was injected under the nitrogen flow and the solution was degassed for another 30 min. After this step, the temperature was raised to 210 °C under a nitrogen atmosphere and 0.03 M TOP-Se$_{0.7}$Te$_{0.3}$ in ODE, prepared in a glovebox under a nitrogen atmosphere, was injected at the rate of 15 mL/h. As the desired growth of the CdSe$_{1-x}$Te$_x$ crown was obtained, the injection of the anion precursor was stopped for 5 min before the subsequent CdSe crown growth. Subsequently, 0.03M ODE-TOP-Se was injected at the rate of 15 mL/h till the desired growth of CdSe was obtained and the injection was stopped for 5 min before the following growth of the CdS crown. The growth of the final CdS crown layer on the obtained CdSe/CdSe$_{1-x}$Te$_x$/CdSe NPLs was

achieved by injecting 0.15 M ODE-S at a rate of 5 mL/h. Finally, the reaction was quenched with a water bath and NPLs were isolated from the undesired species by selective precipitation using hexane as a solvent and ethanol as an anti-solvent. The particles were redispersed and stored in hexane. The growth of each crown layer can be easily followed by the signature peaks of each crown with different elemental compositions in the absorption spectra.

Synthesis of ZnO nanoparticles: 3 mmol zinc acetate dihydrate was dissolved in 30 mL of DMSO solution and stirred vigorously at 1,000 rpm. Then, a mixture of 5.5 mmol TMAH in 10 mL ethanol was injected into the Zn-acetate solution at a rate of 40 mL/h. Under ambient conditions, the mixture was kept stirring for 2 h. Afterward, the ZnO nanoparticles were precipitated by adding ethyl acetate and redispersed in ethanol. To improve the solubility of ZnO nanoparticles, 160 μL of ethanolamine was added to the mixture and kept stirring for 2 h inside the nitrogen filled glovebox. Finally, the resulting nanoparticles washed by addition of ethyl acetate and redispersed in ethanol.

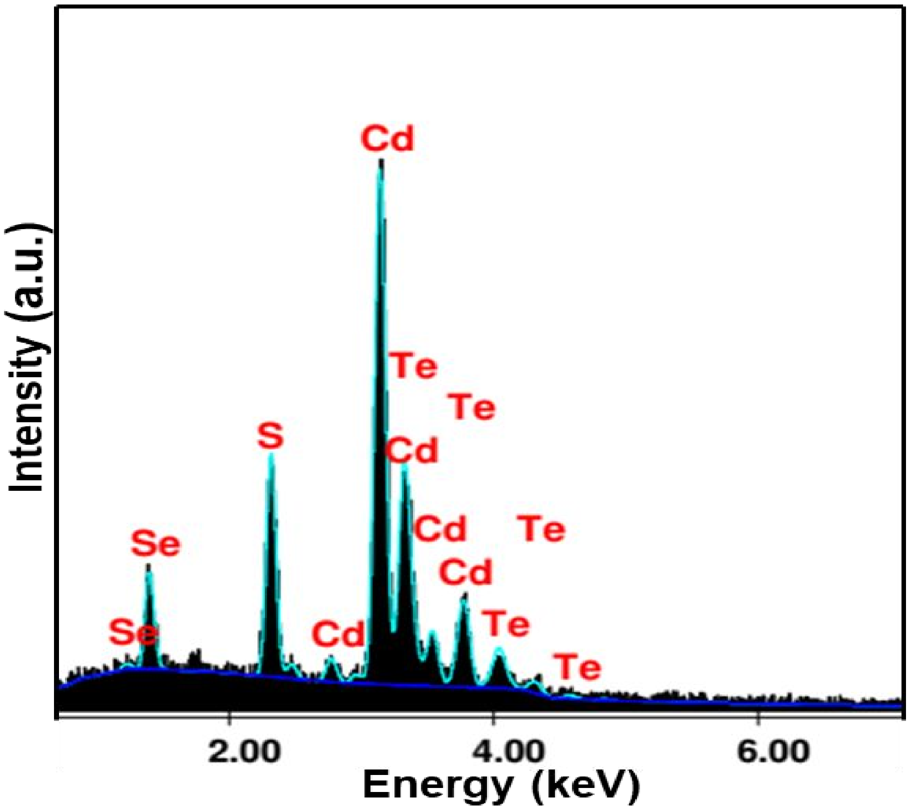

Figure S1. An exemplary scanning electron microscopy with energy dispersive X-Ray (SEM-EDX) spectrum from CdS/CdSe$_{0.44}$Te$_{0.56}$ core/crown NPLs showing the elemental composition. The total atomic percentages of Se, Te and S are 45% while the atomic percentage of Cd is 55%. This is expected from our 4 ML CdS/CdSe$_{1-x}$Te$_x$ core/crown NPLs having 5 monolayers of Cd and 4 monolayers of anions consisting of Se, Te and S atoms.

Table S1. Chemical compositions (x) of CdSe$_{1-x}$Te$_x$ core/crown region for the varied mixture (y) of injected precursor of ODE-TOP-Se$_{1-y}$Te$_y$ obtained by SEM-EDX.

**Injected precursor of ODE-TOP-Se$_{1-y}$Te$_y$** y from the injection composition	**CdS/CdSe$_{1-x}$Te$_x$ core/crown NPLs** x from SEM-EDX
0.30±0.01	0.33±0.01
0.50±0.01	0.56±0.02
0.70±0.01	0.76±0.03

Table S2. Parameters of the numerical fittings (lifetimes, coefficients of each lifetime and their intensity averaged lifetimes) from the TRPL measurements. We employed a bi-exponential decay function for the seed CdS NPLs while for the $CdS/CdSe_{1-x}Te_x$ core/crown NPLs, we used a tri-exponential decay function.

	A_1	τ_1 (ns)	A_2	τ_2 (ns)	A_3	τ_3 (ns)	<τ> (ns)
CdS	181	3.9	936	1.2	--	--	2.2
CdS/CdTe	623	6.1	164	129.1	37.7	1010.2	653.8
$CdS/CdSe_{0.24}Te_{0.76}$	640	5.9	164	90.2	35.3	750.7	471.5
$CdS/CdSe_{0.44}Te_{0.56}$	676	5.8	190	85.7	29.2	797.1	459.3
$CdS/CdSe_{0.67}Te_{0.33}$	853	5.7	171	77.4	25.2	607.8	310.1

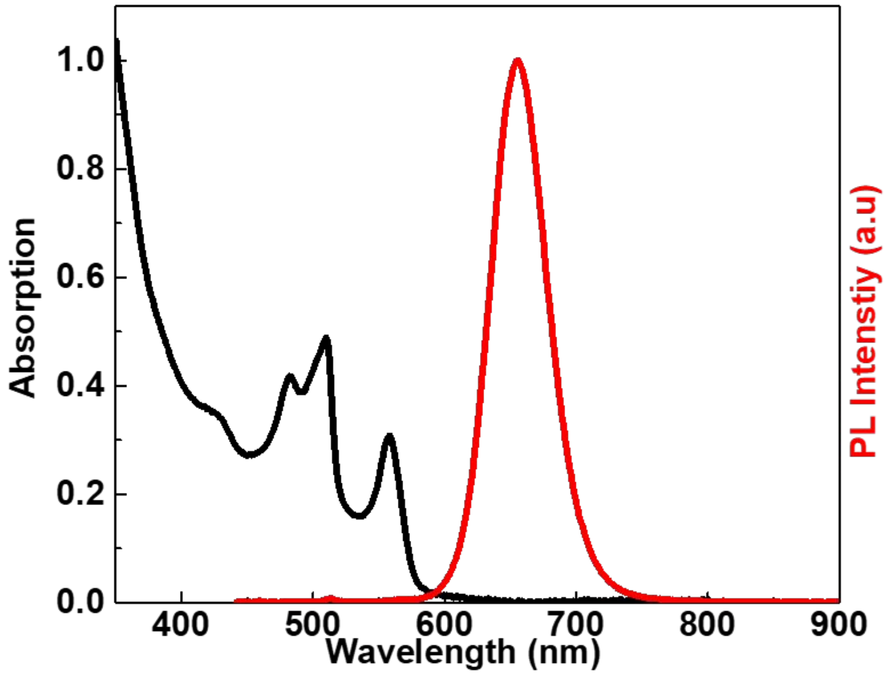

Figure S2. Absorption and PL spectra of the 4 ML CdSe/CdTe NPLs.

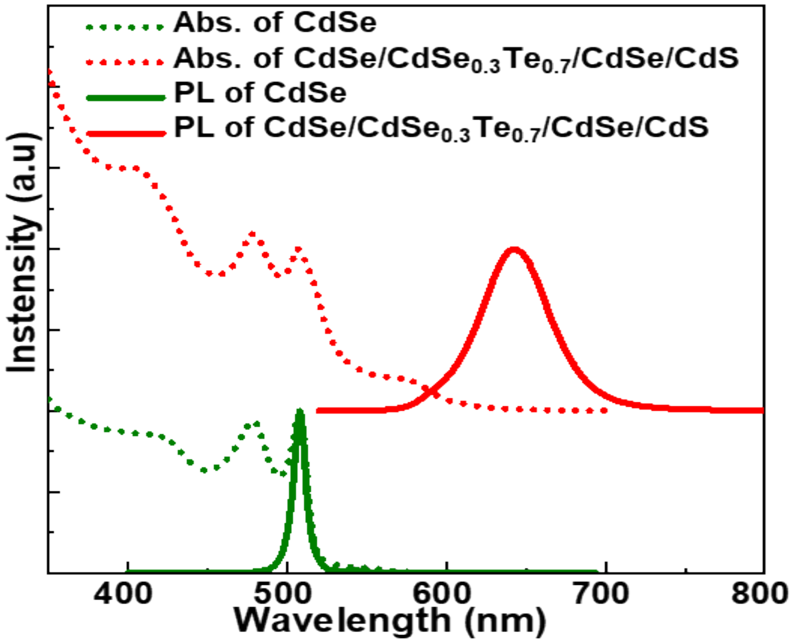

Figure S3. Absorption and PL spectra of the 4 ML CdSe NPLs and $CdSe/CdSe_{0.3}Te_{0.7}/CdSe/CdS$ core/(multi-)crown NPLs. The photoluminescence quantum yield (PLQY) of these $CdSe/CdSe_{0.3}Te_{0.7}/CdSe/CdS$ core/multi-crown NPLs is ~65%, which is lower than the PLQY of $CdSe/CdSe_{0.7}Te_{0.3}/CdSe/CdS$ core/multi-crown NPLs as discussed in the main text.

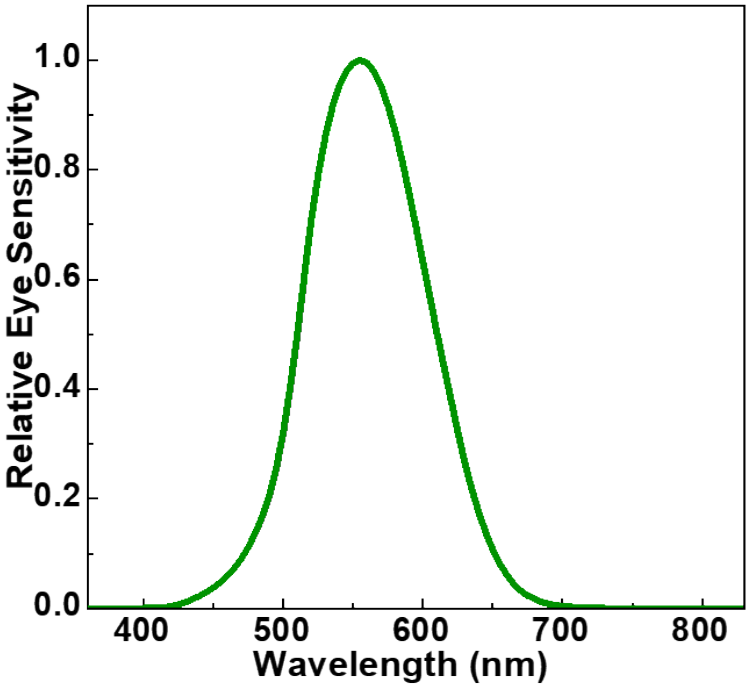

Figure S4. Relative spectral sensitivity of the eye.

Wave function distribution calculations: We calculated the electron and hole wavefunctions in the core/(multi-)crown nanoplatelets considering the problem of a particle-in-a-2D-box and solve the stationary Schrödinger equation, assuming the effective mass approximation, using COMSOL Multiphysics:

$$\left[-\frac{\hbar^2}{2m_{e(h)}}\nabla^2_{e(h)} + V_{e(h)}(x,y)\right]\Psi_{e(h)}(x,y) = E\Psi_{e(h)}(x,y) \qquad \text{(S1)}$$

where $V_{e(h)}(x, y)$ is the potential arising from the conduction (valance) band in the *x*-*y* plane for the electron (hole), $m_{e(h)}$ is the effective mass of the electron (hole), and $\Psi_{e(h)}$ denotes the envelope electron (hole) wavefunction. The electron-hole Coulomb interaction was estimated using the perturbation theory as follows:

$$(H_0 + \lambda V_{Coul})|\psi_n\rangle = E_n|\psi_n\rangle \qquad \text{(S2)}$$

where V_{Coul} is the Coulomb interaction between the electron and the hole, H_0 is the unperturbed Hamiltonian. Using the first-order approximation, the first-order energy correction is given by:

$$E_n^1 = \langle\psi_n^0|V_{Coul}|\psi_n^0\rangle \qquad \text{(S3)}$$

and the new wavefunction, $|\psi_n\rangle$, is given by:

$$|\psi_n\rangle = |\psi_n^0\rangle + |\psi_n^1\rangle = |\psi_n^0\rangle + \sum_{m\neq n}\frac{|\psi_m^0\rangle\langle\psi_m^0|V_{Coul}|\psi_n^0\rangle}{E_n^0 - E_m^0} \qquad \text{(S4)}$$

where $|\psi_n^0\rangle$ are the unperturbed wavefunctions.

In the case of core/crown NPLs, we consider the potential according to Figure S5(a), where V_1 results from the conduction and valence band offsets between CdS and CdTe and V_2 represents the potential offset (2 eV) between the CdTe and ligands. Using the boundary and normalization conditions, the electron and hole wavefunctions were computed.

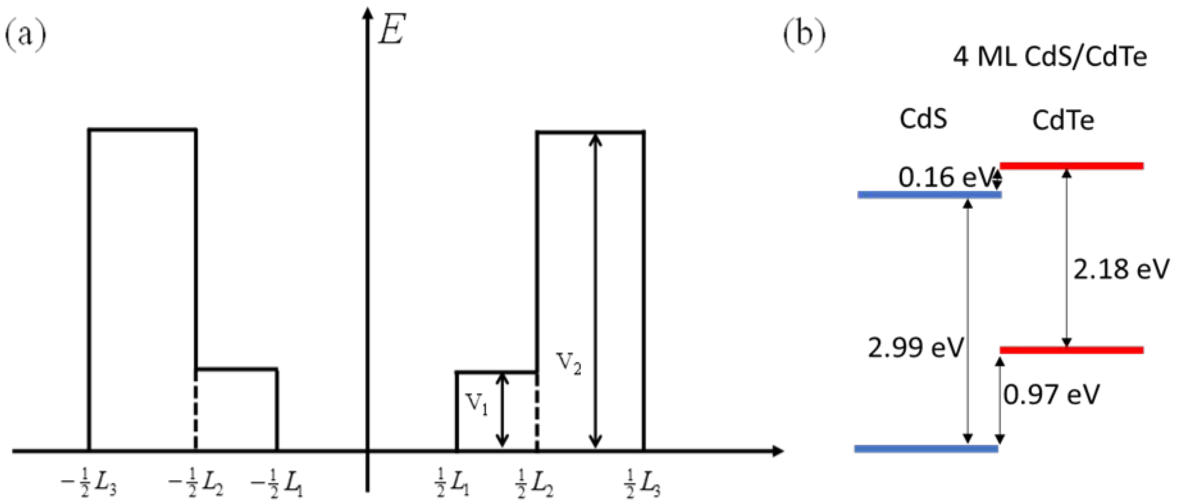

Figure S5. (a) Potential diagram for the electron and hole in the core/crown. V_1 is the potential barrier offset between CdS/CdTe for the electron and hole. V_2 represents the potential barrier offset (2 eV) between the CdTe and ligands. (b) Potential band offsets for the 4 ML CdS/CdTe NPL.

In the case of CdSe/CdSeTe/CdSe/CdS core/multi-crown NPLs, we consider the potentials according to Figure S6, where V_1 results from the conduction and valence band offset between CdSe and CdSeTe, V_2 results from the conduction and valence band offset between CdSe and CdS, and V_3 represents the potential offset (2 eV) between the CdS and ligands. Taking into account the usual boundary and normalization conditions, the electron and hole wavefunctions were calculated.

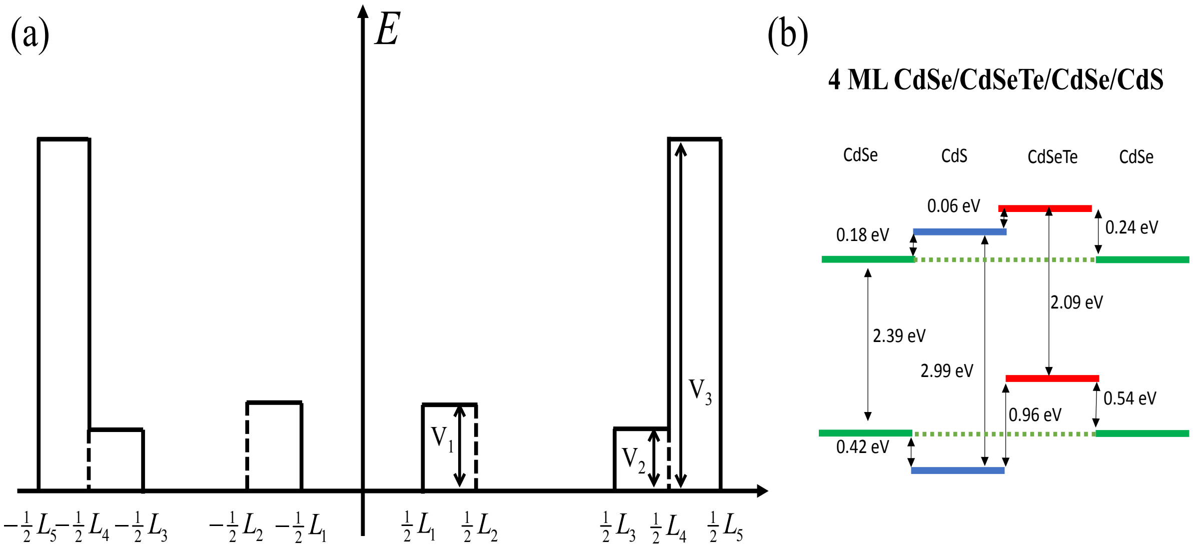

Figure S6. (a) Potential diagram for the electron and hole in the CdSe/$CdSe_{0.7}Te_{0.3}$/CdSe/CdS. V_1 is the potential barrier offset between CdSe and $CdSe_{0.7}Te_{0.3}$ for the electron and hole. V_2 is the potential barrier offset between CdSe and CdS for the electron and hole. V_3 represents the potential barrier offset (2 eV) between the CdTe and ligands. (b) Potential barrier offsets used for the 4 MLs CdSe/$CdSe_{0.7}Te_{0.3}$/CdSe/CdS NPL obtained from Figure 3, 4 and S2.

Numerical Results: Table S3 lists the parameters used for calculating the electron and hole wavefunctions of CdS/CdTe, CdTe/CdS, and CdSe/$CdSe_{0.7}Te_{0.3}$/CdSe/CdS core/(multi)-crown NPLs. First, we discuss the CdS/CdTe core/crown NPLs. Here, in the case of free particle (no Coulomb interactions), electron is confined in the CdS core while hole is confined in the CdTe crown as presented in Figures S7a,b. After including the Coulomb interactions between electron and hole (presented in Figures 4b,c in the main text), the electron wavefunction is redistributed and shaped according to the hole electromagnetic attraction while hole wavefunction is not modified visibly. For the CdTe/CdS core/crown NPLs, similar analysis is undertaken, however in this case, the role of the electron and the hole is interchanged.

Lastly, we discuss CdSe/$CdSe_{0.7}Te_{0.3}$/CdSe/CdS core/multi-crown NPLs. Similarly to the CdS/CdTe case, we consider two cases: the free particle case (no Coulomb interactions) and

the case with electron-hole Coulomb interactions. For the case of no Coulomb interactions (presented in Figures S9a,b), we observed that the electron wavefunction is distributed largely in the CdSe core and partially in the CdSe crown (Figure 4a) while the hole is confined only in the $CdSe_{0.7}Te_{0.3}$ crown due to the band offsets between the CdSe and $CdSe_{0.7}Te_{0.3}$ (Figure S6b). On the other hand, in the case that Coulomb interactions are included (Figures S9c,d), we observed that the electron wavefunction is strongly localized in the CdSe crown and partially in the CdSe core. In contrast, the hole wavefunction is not affected much likely due to the much stronger valence band offset compared to the conduction band offset between the CdSe and $CdSe_{0.7}Te_{0.3}$ interfaces.

Table S3. List of parameters for CdS, CdSe and CdTe used for calculating the electron and hole wavefunctions. m_0 is the electron rest mass. A thickness of 0.35 nm is assumed per monolayer.

Parameters	CdSe	CdS	CdTe
Electron effective mass	0.19 m_0 [3]	0.19 m_0 [3]	0.09 m_0 [3]
Hole effective mass	0.90 m_0 [3]	0.96 m_0 [3]	0.49 m_0 [3]

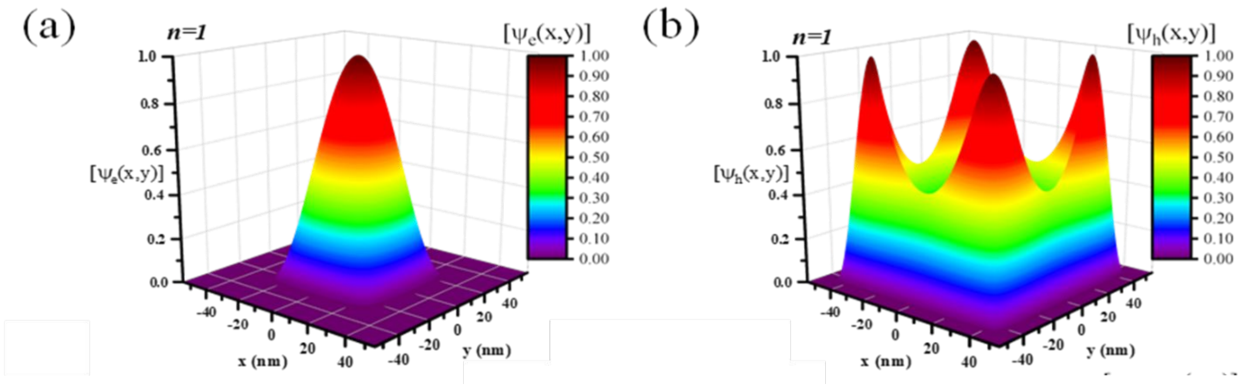

Figure S7. Calculated distribution of (a) electrons and (b) holes wavefunctions in 4 ML CdS/CdTe core/crown NPLs for the free particle case (no Coulomb Interactions).

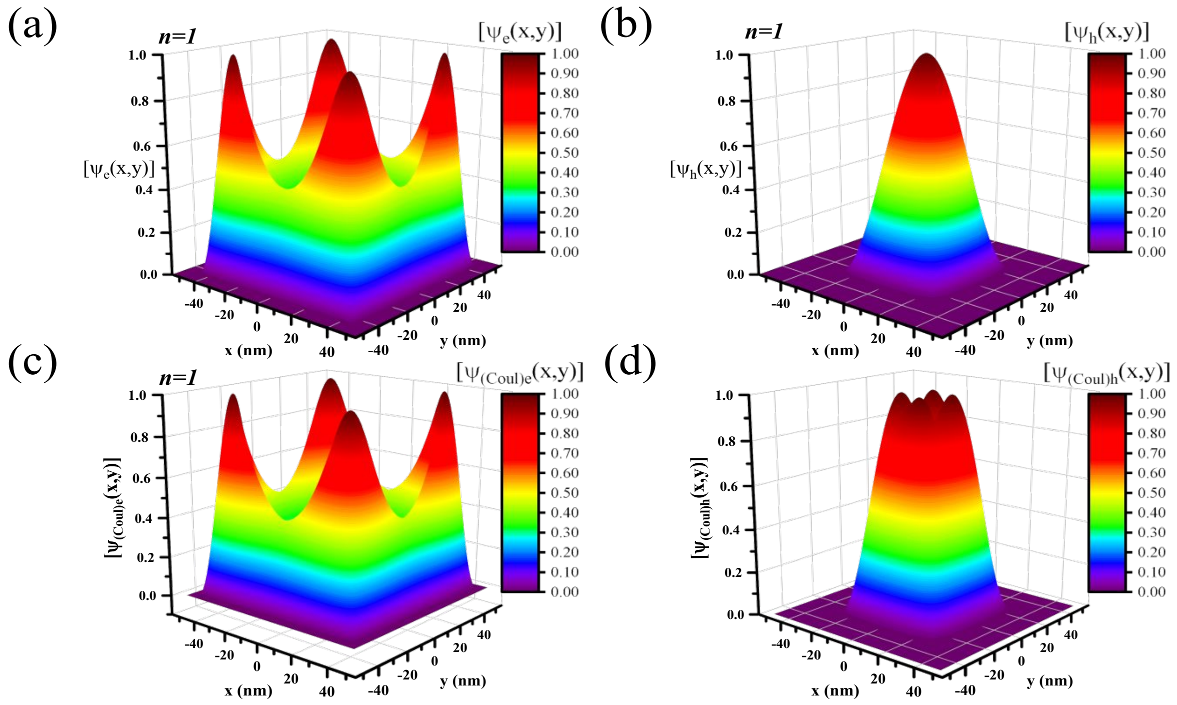

Figure S8. Calculated distribution of (a) electron and (b) hole wavefunctions in 4 ML CdTe/CdS core/crown NPLs for the free particle case (no Coulomb interactions). Calculated distribution of (c) and (d) electron and hole wavefunctions with Coulomb interactions between the electrons and holes in 4 ML CdTe/CdS core/crown NPLs. After including the Coulomb interactions between the electron and the hole, the hole wavefunction is redistributed and shaped according to the electromagnetic attraction while the electron wavefunction is not modified visibly with the introduction of the Coulomb interactions.

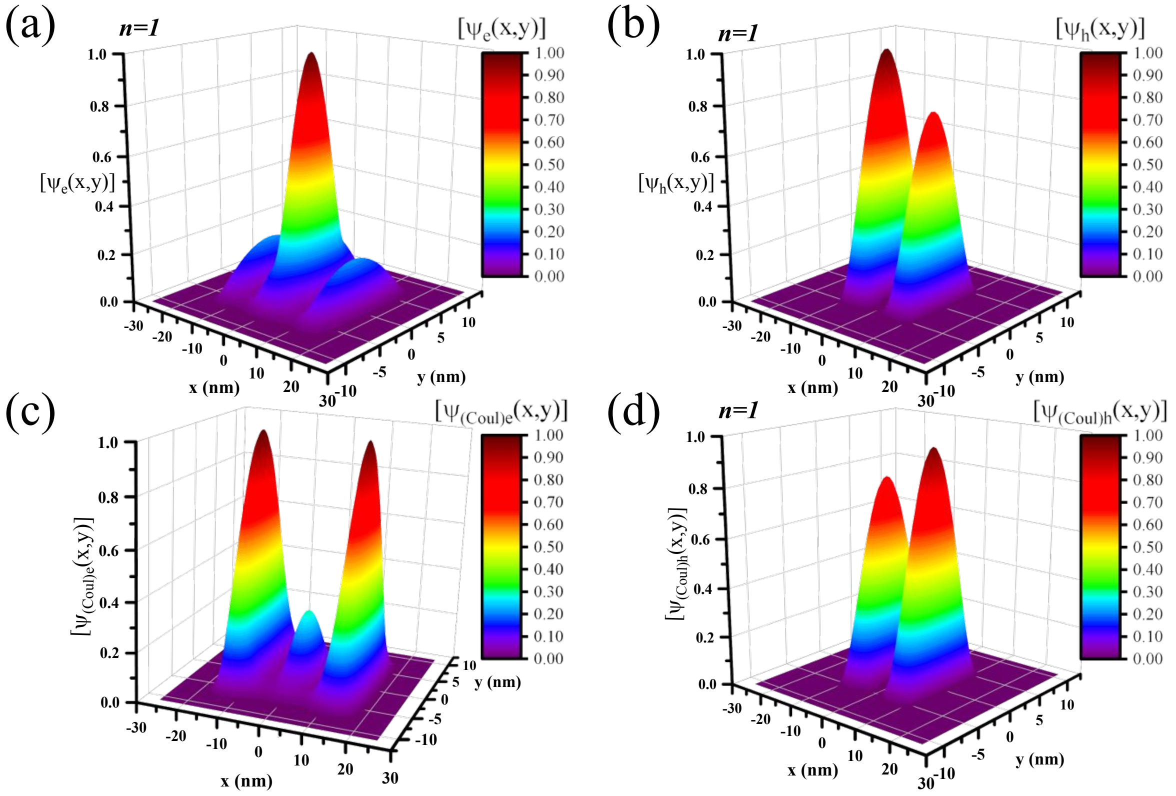

Figure S9. Calculated distribution of (a) electron and (b) hole wavefunctions for the free particle case (no Coulomb Interactions) in 4 ML CdSe/CdSe$_{0.7}$Te$_{0.3}$/CdSe/CdS core/multi-crown NPLs. Calculated distribution of (c) electron and (d) hole wavefunctions with Coulomb interactions in 4 ML CdSe/CdSe$_{0.7}$Te$_{0.3}$/CdSe/CdS core/multi-crown NPLs.

Device fabrication and characterization: The LEDs were fabricated on patterned indium tin oxide (ITO) coated substrates. The substrates were cleaned with detergent, acetone, distilled water, and iso-propanol subsequently, for 10 min each. Then, ITO coated substrates were treated with ozone plasma for 15 min at 30 W to eliminate the residues on the ITO surface and increase the workfunction of ITO. To obtain the LED structure, the PEDOT:PSS solution (filtered through 0.25 μm PTFE membrane filter) was spin-coated onto the ITO substrates at 3,000 rpm for 60 s and baked at 140 °C for 15 min under ambient conditions. The PEDOT:PSS-coated substrates were transferred into a nitrogen-filled glovebox for spin-coating of the poly-TPD layer. Poly-TPD employed as a hole transport layer in our LED. Poly-TPD in

chlorobenzene (8 mg/mL) was spin-coated for 30 s at 2,000 rpm to obtain a thickness of 50 nm and then was baked at 110 °C for 20 min. Afterwards, CdSe/$CdSe_{1-x}Te_x$/CdSe/CdS core/multi-crown NPLs solution (13 mg/mL in toluene) was spin-coated for 45 s at 2,000 rpm and baked at 90 °C for 10 min. For the deposition of the electron transport layer (ETL), ZnO nanoparticles solution in ethanol with a concentration of 25 mg/mL was spin-coated for 60 s at 3,000 rpm to obtain a thickness of 40 nm and baked at 90 °C for 30 min. Finally, the top Al cathode layer (100 nm) was thermally deposited under a base pressure level of 1×10^{-6} Pa. Devices were encapsulated to protect active LED layers from moisture and oxygen exposure before taking the measurements.

EQE values were measured using an integrating sphere (Newport 5.3") coupled with Ocean Optics (QEPro) spectrometer for output light measurements. In the measurements, the devices were put in close contact with the input aperture of the sphere. Current density-voltage-luminance (J-V-L) characteristics of NPL-LEDs were obtained using Agilent Technologies (U3606A) electrometer. A Konica Minolta CS-2000 spectroradiometer was used for the luminance intensity.

Table S4. Maximum EQEs from fabricated LEDs.

Maximum EQEs from each fabricated device	*(%)*
Device 1	9.3
Device 2	8.8
Device 3	9.0
Device 4	8.4
Device 5	7.9
Device 6	8.2
Device 7	7.2
Device 8	7.2
Device 9	6.1
Device 10	7.0
**Average maximum EQE**	**7.9**
**Standard deviation of maximum EQE**	**1.0**

References

[1] M. D. Tessier, P. Spinicelli, D. Dupont, G. Patriarche, S. Ithurria, B. Dubertret, *Nano Lett.* **2014**, *14*, 207.

[2] S. Delikanli, B. Guzelturk, P. L. Hernández-Martínez, T. Erdem, Y. Kelestemur, M. Olutas, M. Z. Akgul, H. V. Demir, *Adv. Funct. Mater.* **2015**, *25*, 4282.

[3] S. Ithurria, M. D. Tessier, B. Mahler, R. P. S. M. Lobo, B. Dubertret, A. L. Efros, *Nat. Mater.* **2011**, *10*, 936.
